# Hypertriglyceridemia Induced Pancreatitis (Chylomicronemia Syndrome) Treated with Supportive Care

**DOI:** 10.1155/2014/767831

**Published:** 2014-11-23

**Authors:** Emin Uysal, Yahya Ayhan Acar, Emel Gökmen, Ahmet Kutur, Hatice Doğan

**Affiliations:** ^1^Department of Emergency Medicine, Bagcilar Training and Research Hospital, Bağcılar, 34200 Istanbul, Turkey; ^2^Department of Emergency Medicine, Etimesgut Military Hospital, Etimesgut, 06797 Ankara, Turkey; ^3^Department of Internal Medicine, Bagcilar Training and Research Hospital, Bağcılar, 34200 Istanbul, Turkey

## Abstract

Hypertriglyceridemia is a rare cause of pancreatitis. In treatment pancreatic rest, lifestyle changes, medications (fibrates, n-3 polyunsaturated fatty acids, and nicotinic acid) are essential. Many experimental treatment modalities have been reported as insulin and heparin infusion and plasmapheresis. In this study we present the hypertriglyceridemia-induced pancreatitis treated with supportive care.

## 1. Introduction

Hypertriglyceridemia-induced pancreatitis (HTIP) is a rare but well known clinical condition. Triglyceride (TG) level above 1000 mg/dL is defined as chylomicronemia and chylomicronemia syndrome (CS) is the condition being TG > 1000 mg/dL and additionally one of eruptive xantomas, lipemia retinalis, or abdominal pain/pancreatitis [1]. CS is also HTIP and it was defined for the practical approach to management [1]. The exact mechanism of hypertriglyceridemia (HTG) in pancreatitis could not be identified clearly [2] and many treatment modalities were reported. In this study, we reported a HTIP (chylomicronemia syndrome) case treated with supportive care.

## 2. Case Presentation

A 42-year-old man was admitted to emergency department with a 1 h history of abdominal pain, nausea, and nonbilious vomiting. He had a past medical history of diabetes mellitus, hypertension, hypothyroidism, and hypertriglyceridemia. The patient reported taking levothyroxine (50 mg oral once a day), amlodipine (5 mg oral once a day), insulin glargine (20 U subcutaneous once a day), metformin (1000 mg oral three times a day), and rosuvastatin (20 mg oral once a day). He ran out of fenofibrate the month before. The patient denied any fever, jaundice, or alcohol consumption. The physical examination showed a temperature of 36.8°C, blood pressure of 130/80 mmHg, respiratory rate of 18/min, and pulse rate of 110/min. The abdomen examination was significant for rebound and tenderness in epigastric region. Any other abnormality was not detected in abdominal, respiratory, cardiovascular, and neurologic examination.

**Figure 1:**
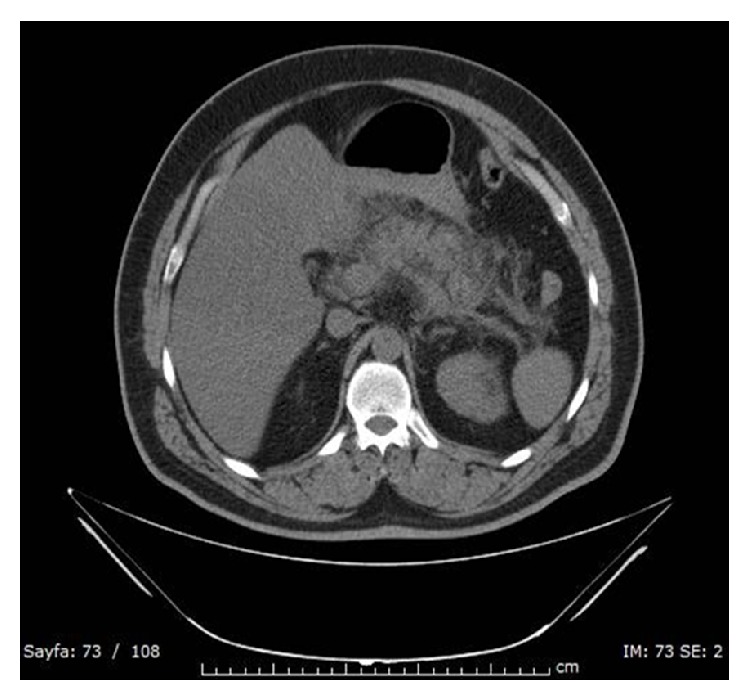
Computed tomography of abdomen showed edematous characteristics in peripancreatic region.

Relevant laboratory results at the time of admission were as follows. White blood cell count was 13.740/mm^3^ (ref: 3.2–9.7), hemoglobin was 17.5 g/dL (ref: 13–17.2), and C-reactive protein was 1.4 mg/dL (ref: 0–5). Blood glucose level was 417 mg/dL (ref: 74–106), urine ketones were (+) and in arterial blood gas analyze pH was 7.44, pO2: 92.6, pCO2: 34.8, and SaO2: 99.1%. While serum amylase was normal (75 U/L, ref: 30–118), lipase level was elevated (2914 U/L, ref: 6–51). Transaminase levels were also elevated (ALT: 157 U/L, ref: 1–40; AST: 17, ref: 1–40). Measured serum sodium was 116 mmol/L (ref: 132–136), and corrected serum sodium was 124 mmol/L [3]. Chest X-ray and abdominal X-ray were normal. USG of the abdomen showed grade 3 hepatosteatosis but any abnormality in biliary or pancreatic regions was not reported. Abdominal computed tomography (CT) confirmed pancreatic edema consistent with acute pancreatitis (Figure 1).

**Figure 2:**
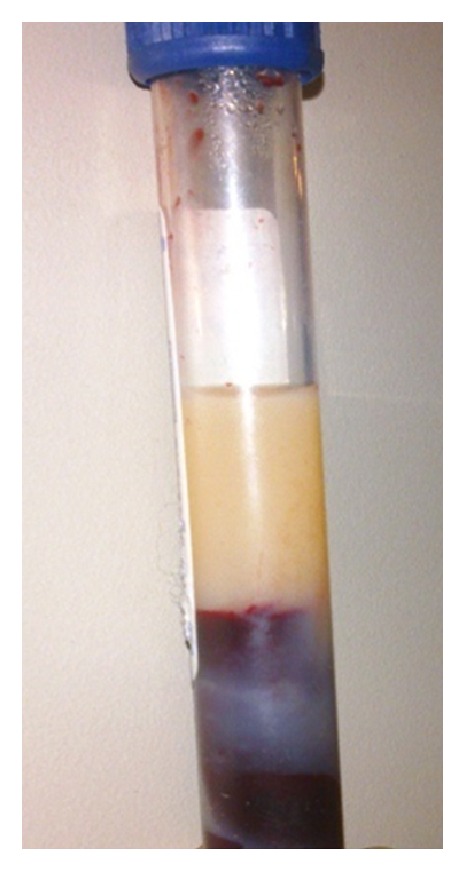
Lipemic serum of patient.

Patient was hospitalized in Internal Medicine Clinic with the diagnosis of nonbiliary pancreatitis. The patient had uncontrolled diabetes mellitus and hyperlipidemia (HbA1c: 7.3%, ref: 4–6; glucose: 352 mg/dL, ref: 74–106; cholesterol: 800 mg/dL, ref: 1–200; triglyceride: 3454 mg/dL, ref: 0–200) and diagnosis was directed to HTIP. The serum was lipemic on gross examination (Figure 2). The patient was kept nil per mouth, intravenous fluid therapy, and oral medical therapy (including fenofibrate) started consistent with previous therapy. During the hospital stay, his abdominal pain, triglyceride, and pancreatic enzyme levels were improved.

He had no abdominal pain and was discharged from hospital on day 9 with a triglyceride level of 1669 mg/dL, cholesterol level of 435 mg/dL, amylase level of 40 U/L, lipase level of 91 U/L, and sodium level of 136 mg/dL. HTIP did not recur in six-month follow-up.

## 3. Discussion

Pancreatitis is a clinical condition characterized with broad inflammation in pancreas. Although biliary stones and alcohol consumption are the major etiologic group of pancreatitis, HTG is a rare but well-known cause of pancreatitis in up to 10% of all cases [4]. Causes of HTIP can be divided into two main groups: (1) genetic factors: familial combined hyperlipidemia, familial hypertriglyceridemia, familial dysbetalipoproteinemia, and familial chylomicronemia syndrome and (2) secondary factors: untreated/poorly controlled diabetes mellitus (DM), alcohol abuse, pregnancy, and medications [5]. Possible mechanism of pancreatitis in hypertriglyceridemic patients is the damage of asinar cells and microvascular membrane due to excessive free fatty acid and lysolecithin formation in pancreatic bed from lipoprotein substrates [6].

Initial treatment of HTIP includes pancreas rest (by limiting oral intake, aggressive intravenous hydration, and analgesia) [5]. For further treatment of HTIP, plasmapheresis [7–9], heparin infusion [10–16], and subcutaneous heparin [17] were reported, but still they are considered as experimental treatment modalities in HTIP [5]. Apheresis has been recommended as category III (optimum role of apheresis therapy is not established; individualized decision is necessary) and grade-2C (weak recommendation, low-quality, or very low-quality evidence) for hypertriglyceridemic pancreatitis by American Society for Apheresis [18]. European Atherosclerosis Society (EAS) mentioned apheresis to be able to lower TG levels rapidly in acute settings rather than a standard therapy [19]. Heparin has been reported as controversial because of the hemorrhage into the pancreatic bed in the setting of pancreatitis [18]. Anderson et al. reported standard therapy of intravenous fluids, nil by mouth and supportive care alone was equivalent to the use of dextrose and insulin in resolution of HTG in pancreatitis [20]. Our patient was treated with supportive care, but previous insulin glargine therapy was continued for DM. We report a HTIP case treated with supportive care without any administration of heparin or apheresis [21].

It was reported that TG levels below 1772 mg/dL (20 nM) are unlikely to be the primary cause of pancreatitis and most of these patients have also uncontrolled diabetes mellitus as secondary cause [2]. However, TG levels > 1000 mg/dL are considered as a causative reason for HTIP recurrence; this threshold is still arbitrary [4]. Hospitalization and nothing by mouth were recommended for patients whose TG levels > 500 mg/dL with abdominal pain [1]. Our patient's TG level was 3454 mg/dL in admission and 1669 mg/dL in discharge. We discharged the patient because of improving clinical condition, lack of abdominal pain, decreasing trend in TG levels, good compliance to treatment, and diet.

Serum amylase concentration may be in normal ranges in HTIP [11]. Our patient's amylase levels were also in normal ranges. Mechanism of normoamylasemia is believed to be the inadequacy of calorimetric method and serial dilutions of the sample can reduce interference of light transmission by hyperlipidemic serum [22, 23]. Our patient had normal amylase levels and high lipase levels. Lipase levels may be more sensitive in pancreatitis diagnosis or minimally affected from HTG state.

Clinical suspicion of pancreatitis should be kept in mind even with normal amylase levels especially in the presence of hypertriglyceridemia. Supportive care may be sufficient in therapy. Controlled clinical trials are essential for improving therapy modalities in HTIP.

## Prior Publication

This article has not been published or submitted for publication elsewhere, in whole or in part, before submission to the Case Reports in Critical Care.
